# What Is Abnormal in Normal Karyotype Acute Myeloid Leukemia in Children? Analysis of the Mutational Landscape and Prognosis of the TARGET-AML Cohort

**DOI:** 10.3390/genes12060792

**Published:** 2021-05-21

**Authors:** Morten Krogh Herlin, Sara A. Yones, Eigil Kjeldsen, Linda Holmfeldt, Henrik Hasle

**Affiliations:** 1 Department of Pediatrics and Adolescent Medicine, Aarhus University Hospital, 8200 Aarhus N, Denmark; henrik.hasle@clin.au.dk; 2 Department of Clinical Medicine, Aarhus University, 8200 Aarhus N, Denmark; 3 Science for Life Laboratory, Department of Cell and Molecular Biology, Uppsala University, 75185 Uppsala, Sweden; sara.younes@icm.uu.se; 4 Department of Hematology, Aarhus University Hospital, 8200 Aarhus N, Denmark; eigil.kjeldsen@clin.au.dk; 5 Department of Immunology, Genetics, and Pathology, Science for Life Laboratory, Uppsala University, 75185 Uppsala, Sweden; linda.holmfeldt@igp.uu.se

**Keywords:** pediatric acute myeloid leukemia, normal karyotype, cytogenetically normal, mutational landscape, molecular genetics, cytogenetics, cancer genetics, diagnosis, survival, prognosis

## Abstract

Normal karyotype acute myeloid leukemia (NK-AML) constitutes 20–25% of pediatric AML and detailed molecular analysis is essential to unravel the genetic background of this group. Using publicly available sequencing data from the TARGET-AML initiative, we investigated the mutational landscape of NK-AML in comparison with abnormal karyotype AML (AK-AML). In 164 (97.6%) of 168 independent NK-AML samples, at least one somatic protein-coding mutation was identified using whole-genome or targeted capture sequencing. We identified a unique mutational landscape of NK-AML characterized by a higher prevalence of mutated *CEBPA*, *FLT3*, *GATA2*, *NPM1*, *PTPN11*, *TET2*, and *WT1* and a lower prevalence of mutated *KIT*, *KRAS*, and *NRAS* compared with AK-AML. Mutated *CEBPA* often co-occurred with mutated *GATA2*, whereas mutated *FLT3* co-occurred with mutated *WT1* and *NPM1*. In multivariate regression analysis, we identified younger age, WBC count ≥50 × 10^9^/L, *FLT3*-internal tandem duplications, and mutated *WT1* as independent predictors of adverse prognosis and mutated *NPM1* and *GATA2* as independent predictors of favorable prognosis in NK-AML. In conclusion, NK-AML in children is characterized by a unique mutational landscape which impacts the disease outcome.

## 1. Introduction

Acute myeloid leukemia (AML) is a heterogenous malignant disease of the bone marrow resulting from the accumulation of acquired somatic genetic lesions in myeloid progenitor cells [1]. Overall survival of childhood AML has reached ~70% based on intensive chemotherapy regimens with relapse still occurring in ~30% of all patients [2], and therefore novel targeted therapeutics are warranted to increase cure rate [3]. Genetic characterization of AML is a hallmark both in research unravelling the disease biology and identifying new therapeutic targets, as well as in the clinic supporting disease classification, risk stratification, disease monitoring and therapy guidance [4]. Disease classification is, however, still mainly based on the identification of recurrent chromosomal lesions [5] from routine cytogenetic examinations. These cytogenetic examinations involve conventional chromosomal banding analysis and fluorescence in situ hybridization (FISH) targeting recurrent cytogenetic lesions including gene fusions, as well as reverse transcriptase-polymerase chain reaction (RT-PCR) targeting recurrent gene fusion transcripts and common gene mutations (e.g., *FLT3*-internal tandem duplication [ITD]) [6].

Importantly, ~20–25% of pediatric AML show no cytogenetic abnormalities following routine cytogenetic examination, and this group is referred to as normal karyotype or cytogenetically normal AML (NK-AML) [7,8,9,10]. Molecular analysis by RT-PCR targeting selected genes such as *FLT3*, *NPM1*, and *CEBPA* is useful in these cases to identify prognostically important mutations [11,12,13,14]. These aberrations do, however, only represent a limited picture of the genetics in NK-AML. Fortunately, the advent of Next-Generation sequencing (NGS) technologies has allowed more detailed genetic investigations of AML through targeted gene panel sequencing, whole exome sequencing, whole-genome sequencing (WGS), and transcriptomic RNA sequencing. Using these techniques, major efforts have been made to unravel the molecular landscape of pediatric AML and disease progression [10,15,16] including a study of focusing on normal karyotype *FLT3*-ITD positive AML [17].

From the publicly available data from the Therapeutically Applicable Research to Generate Effective Treatments (TARGET) initiative AML study (TARGET-AML) [10], we here aimed to characterize the molecular landscape and identify determinants of outcome in childhood NK-AML.

## 2. Materials and Methods

### 2.1. Data Source

The present study was based on the publicly available data from the TARGET-AML initiative [10]. The original clinical and genetic data can be accessed at https://target-data.nci.nih.gov/Public/AML/ (accessed on 24 January 2021).

### 2.2. Study Population

The AML patient samples in the TARGET-AML study were obtained from the Children’s Oncology Group protocols AAML0531 [18], AAML03P1 [19], and CCG-2961 [20]. The TARGET-AML study profiled a total of 815 children and young adults for somatic mutations at diagnosis (based on paired leukemia-remission samples) including WGS of 197 subjects as well as targeted capture sequencing (TCS) averaging 500× coverage in 800 subjects used for validation [10]. For our analysis, we identified the study cohort combining the clinical data files of the discovery and validation cohorts (date version: 13 December 2018).

### 2.3. Genetic Data

Cytogenetic data including International System for Human Cytogenetic Nomenclature (ISCN) karyotypes used for disease classification were obtained from the clinical data files.

For mutational status in each sample, we used the spreadsheets reporting protein-coding somatic variants identified using WGS (n = 197) and TCS (n = 684) (Supplementary Tables S5 and S6 in Bolouri et al. [10]). For survival analyses, we also included mutational status from routine testing of *FLT3*, *NPM1*, *CEBPA*, and *WT1* provided in the clinical data. For mutational burden of single nucleotide variants (SNVs) and insertions and deletions (indels) in each sample, we used the spreadsheet reporting variant calls from Strelka and SAMtools mpileup variant callers (Supplementary Table S4a in Bolouri et al. [10]). Our SNV/indel analysis depended on the accuracy of these processed variant data and we did not apply any additional variant filtering.

### 2.4. Genetic and Statistical Analysis

Based on the cytogenetic information provided in the clinical data, samples were categorized as core-binding factor AML (CBF-AML) including t(8;21)(q22;q22) and inv16(p13.1q22), *KMT2A* (*MLL*) rearranged AML, another abnormal karyotype AML and NK-AML. The first three groups are collectively referred to as abnormal karyotype AML (AK-AML) in this study. The assigned categorization based on ISCN karyotypes remained throughout all analyses in this study.

Recurrently mutated genes (mutated in ≥2 samples) were identified in NK-AML. The SNV/indel mutational burden in NK-AML vs AK-AML was plotted and tested for equal distributions using the Wilcoxon rank sum test. The SNV/indel mutational landscapes of NK-AML vs AK-AML were visualized using two-level doughnut charts [21], considering all genes mutated in ≥2% of the samples within both groups with all genes categorized according to The Cancer Genome Atlas (TCGA) functional groups [22]. Mutually exclusive and co-occurring mutation events between genes in NK-AML vs. AK-AML were identified using Maftools (v2.2.10) [23], which uses pairwise Fisher’s exact test on a 2 × 2 contingency table containing frequencies of mutated and non-mutated samples. Computed odds ratios indicated either co-occurrence or mutual exclusivity.

Baseline patient characteristics and mutation frequencies were summarized using descriptive statistics. For comparing NK-AML vs AK-AML characteristics Wilcoxon rank sum test was used for continuous variables and χ^2^ test for categorical variables including mutation status. Event-free survival (EFS; events defined as induction failure, induction death, death in first complete remission, or relapse) and overall survival (OS) with 95% confidence intervals (CIs) were computed using the Kaplan-Meier estimator. Cumulative incidence of relapse (CIR) was estimated using the pseudo values method with death as competing risk [24]. Log rank tests were used for comparison of different EFS and OS distributions by cytogenetic group, patient characteristics, and mutational status. Cox proportional hazards regression was used to compute hazard ratios as a measure of the relative risk of any event or death during follow-up in univariate analysis as well as multivariate analysis adjusted for baseline risk factors, *FLT3-*internal tandem duplication (ITD), and mutational status of other co-occurring genes as relevant. Stratified analyses included cytogenetic group for the entire study cohort and for the NK-AML cohort, baseline characteristics and mutational status for genes mutated in ≥10 samples with wildtype NK-AML patients as reference. Stem cell transplantation was excluded from the analysis due to no time-points provided in the clinical data necessary for its inclusion in the model as a time-dependent variable. The assumption of proportional hazards was verified using Schoenfeld residuals test and we checked for multicollinearity computing variance inflation factor and tolerance values. Significance level was set at *p <* 0.05.

Data management and analysis of the genetic and clinical data were performed using Stata Statistical Software Package (v14.2; StataCorp. LP, College Station, TX, USA). Analysis of co-occurrence and mutual exclusivity was performed in *R* (v3.6.2) using the Maftools library (v.2.2.10).

## 3. Results

### 3.1. Study Cohort and Clinical Characteristics

From the TARGET-AML study cohort of 966 patients, we excluded patients with no clinical data available (n = 31), aged 20 years or above (n = 16), French-American-British classification (FAB) M3 morphology (n = 2), and no cytogenetic information (n = 44), to obtain the final study cohort 873 patients in this analysis. We identified 208 (24%) NK-AML and 665 (76%) AK-AML patients (CBF-AML, n = 248; *KMT2A* rearranged, n = 175; other AK-AML, n = 242). Table 1 summarizes the patient characteristics stratified by cytogenetic group. Patients with NK-AML were characterized by older age at diagnosis (median: 12 years, *p* < 0.001) which has previously been reported [25], a high prevalence of FAB-M1 morphology (*p* < 0.001), and a high frequency of stem cell transplantation (SCT) in first complete remission (CR1) (*p* < 0.001) compared to AK-AML. No significant differences were seen in 5-year EFS and OS. However, 5-year CIR in NK-AML was lower compared with *KMT2A* rearranged and other AK-AML (Table 1).

### 3.2. Mutational Landscape of NK-AML

AML samples from 661 of 873 (76%) patients were analyzed by WGS and/or TCS including samples from 168 of 208 (81%) NK-AML patients (Table 1). Of the sequenced NK-AML samples, 39 samples were examined by WGS of which 32 samples were validated by TCS. In addition, 129 samples were analyzed by TCS alone (SNV/indel detection only). From samples analyzed by both WGS and TCS, mutational events were only counted once. We compared the baseline characteristics of sequenced and non-sequenced NK-AML patients and found an older age distribution among patients with sequenced samples (median: 13 years, *p* = 0.004). This may reflect a higher amount of bone marrow aspirate acquired from older patients, needed for sequencing studies.

In all sequenced NK-AML samples, a total of 593 gene mutations including SNVs, indels, copy number variants (CNVs, i.e., focal deletions/duplications) and gene fusions affecting 135 different genes were identified in samples from 164/168 (97.6%) patients (see Supplemental Table S1 for an overview of the mutation status of all NK-AML samples). A total of six gene fusions not identified in the cytogenetic workup were identified including *KMT2A-MLLT4*, *KMT2A-MLLT3*, *HNRNPH1-ERG*, *NUP98-NSD1*, *CBFA2T3-GLIS2*, and *SEPT6-KMT2A*. CNVs were identified in samples from eight patients with *ZEB2* as the most commonly impacted gene (n = 4) (Supplemental Table S1). In four NK-AML samples, all analyzed by WGS, no mutations were identified. No specific characteristics were found in these patients and they all varied in terms of blast percentages, white blood cell (WBC) count, and age group.

**Figure 1:**
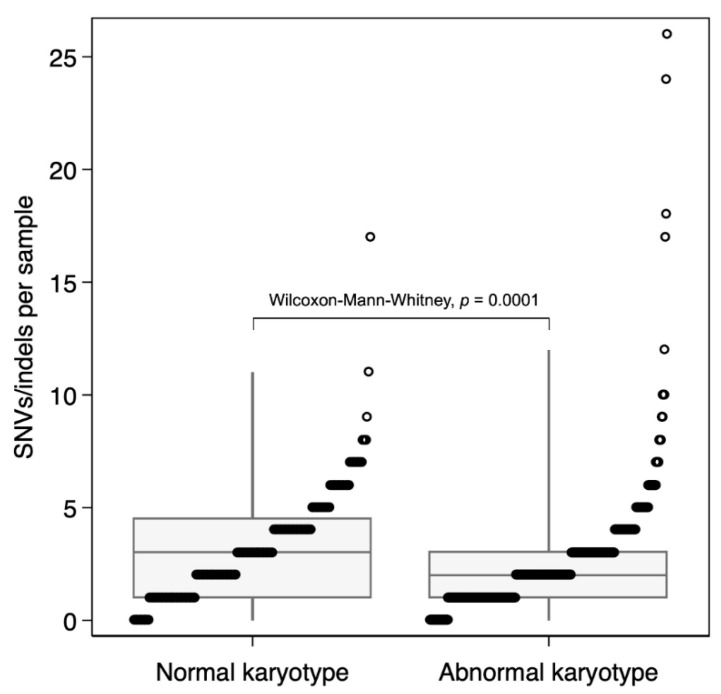
Single nucleotide variant (SNV)/indel mutational burden in pediatric normal vs. abnormal karyotype AML. Total number of SNVs and indels in each sample. The boxplots show the median and interquartile ranges (box) with the 1st and 99th percentiles (whiskers).

A higher SNV/indel count was identified in NK-AML compared with AK-AML (*p* = 0.0001) with a median of 3 called SNVs/indels in NK-AML (range 0–17, Figure 1). This is, however, overall, still a very low mutational burden compared to other cancers, as reported by Bolouri et al. [10].

**Figure 2:**
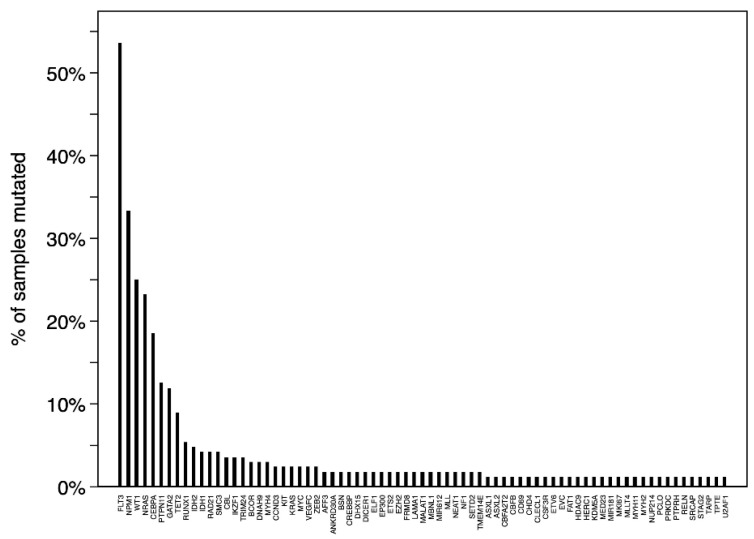
Recurrently mutated (≥2 samples) genes in pediatric normal karyotype AML.

Figure 2 shows all 74 recurrently mutated genes (mutated in ≥2 samples) with *FLT3* (90/168, 54%), *NPM1* (56/168, 33%), *WT1* (42/168, 25%), *NRAS* (39/168, 23%), and *CEBPA* (31/168, 18%) as the top five most commonly mutated genes in NK-AML. We found significantly higher prevalence proportions of *FLT3* (*p* < 0.001), *NPM1* (*p* < 0.001), *WT1* (*p* < 0.001), *CEBPA* (*p* < 0.001)*,* GATA2 (*p* < 0.001), *PTPN11* (*p* = 0.01), and *TET2* (*p* = 0.006) mutants in NK-AML compared with AK-AML, whereas mutated *NRAS* (*p* = 0.05), *KIT* (*p* < 0.001), and KRAS (*p* < 0.001) were more rarely detected in NK-AML. Mutated *NPM1* was associated with older age (median: 14 years, range: 3–20, *p* = 0.009) in NK-AML.

**Figure 3:**
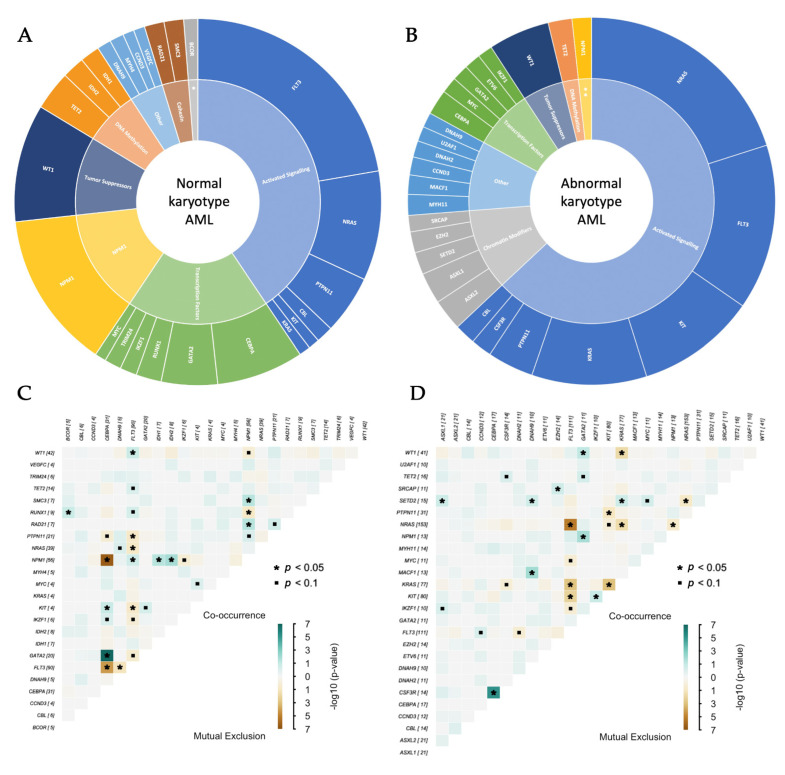
(**A**,**B**) The mutational landscapes of pediatric normal karyotype (**A**) vs abnormal karyotype (**B**) AML. (**C**,**D**) Co-occurrence and mutual exclusion of mutations in commonly altered genes of pediatric normal karyotype (**C**) vs abnormal karyotype (**D**) AML. The panels include genes mutated in ≥2% of samples within the respective groups. * Chromatin modifiers; ** *NPM1*.

Figure 3A,B depict the mutational landscapes of NK-AML and AK-AML of genes mutated in ≥2% of samples in the respective group. Mutations in these genes represented 77% and 64% of all SNV/indels identified in NK-AML and AK-AML, respectively. The NK-AML mutational landscape was characterized by a diversity in implicated TCGA functional groups, including activated signaling (41%), transcription factors (19%), *NPM1* (14%), tumor suppressors/*WT1* (10%), and DNA methylation (7%). In comparison, the AK-AML mutational landscape mostly included genes involved in activated signaling (63%), chromatin modification (11%), and transcription factors (8%).

Next, we investigated the mutual exclusive and co-occurring gene pairs in NK-AML and AK-AML, considering genes mutated in ≥2% of samples in their respective groups (Figure 3C,D). Of the most commonly mutated genes in NK-AML, we identified mutated *CEBPA*/*GATA2* (*p* = 1.3 × 10^−7^), *FLT3*/*WT1* (*p* = 0.03), and *FLT3*/*NPM1* (*p* = 0.045) as commonly co-occurring events, whereas mutated *CEBPA* was likely to be mutually exclusive of both mutated *NPM1* (*p* = 3.0 × 10^−7^) and *FLT3* (*p* = 2.9 × 10^−5^).

### 3.3. Mutational Status and Prognosis in NK-AML

**Figure 4:**
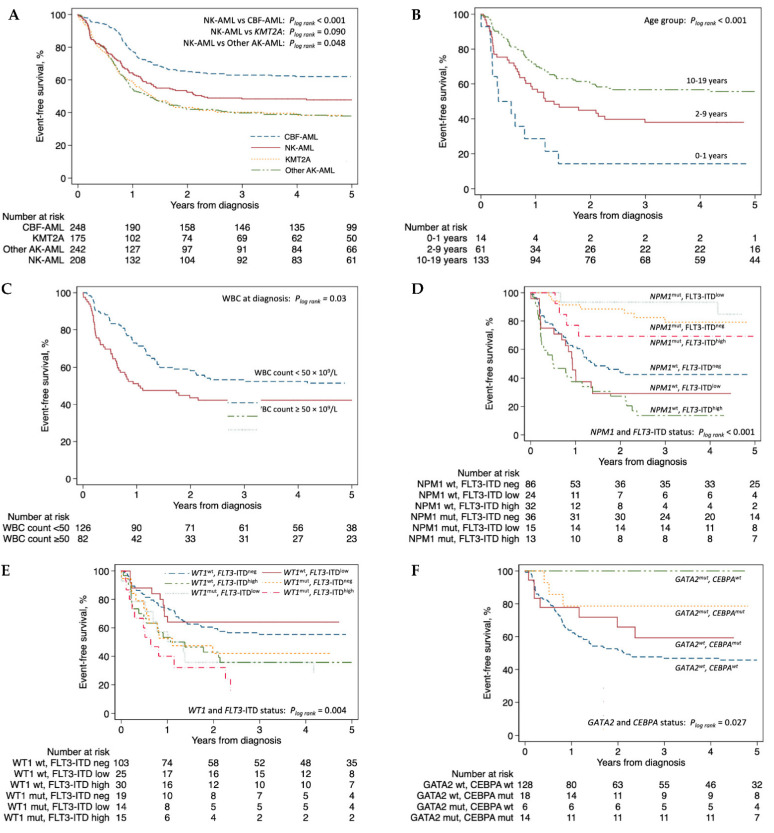
Kaplan-Meier curves of event-free survival by (**A**) cytogenetic group (entire TARGET-AML cohort), (**B)** age group, (**C**) WBC count at diagnosis, (**D**) *NPM1* and *FLT3*-ITD status, (**E**) *WT1* and *FLT3*-ITD status, (**F**) *CEBPA* and *GATA2* status. Panels (**B**–**F**) are restricted to normal karyotype AML patients alone. Abbreviations: AK-AML, abnormal karyotype AML; CBF-AML, core-binding factor AML; ITD, internal tandem duplication; KMT2A, *KMT2A* rearranged AML; mut, mutation; neg, negative; NK-AML, normal karyotype AML; pos, positive; WBC, white blood cell; wt., wildtype.

Median follow-up time for non-deceased NK-AML patients was 5.5 years (range 0.3–10.9). We investigated EFS using Kaplan-Meier curves stratified by selected variables with log rank test results (Figure 4). Additional curves for EFS and OS with log rank test results are provided in Supplemental Figures S1 and S2, respectively. NK-AML was associated with intermediate risk with a 5-year EFS of 48% (95% CI: 41–54%), inferior to CBF-AML (62%; CI: 56–68%), but superior compared to *KMT2A* rearranged AML (38%; CI: 31–46%) and other AK-AML (38%; CI: 32–44%) (Figure 4A). Five-year OS in NK-AML was 62% (95 CI: 55–69%) compared to CBF-AML (80%; CI: 74–85%), *KMT2A* (58%; CI: 50–65%) and other AK-AML (50%; 55–69%) (Figure S2A).

In NK-AML, younger age at diagnosis was associated with inferior EFS (*p* < 0.001; Figure 4B). Furthermore, WBC count ≥50 × 10^9^/L was also associated with inferior EFS (*p* = 0.03; Figure 4C). *FLT3*-ITDs were associated with inferior 5-year EFS in NK-AML (40%; CI: 30–51%; Figure S1D). Considering the *FLT3*-ITD allelic ratio (AR), co-occurrence of a *FLT3*-ITD of low AR (<0.5) with mutated *NPM1* was associated with a favorable outcome (5-year EFS: 85%; CI 51–96%) whereas FLT3-ITD of high AR (≥0.5) with mutated *NPM1* was associated with intermediate risk (5-year EFS: 69%; CI: 37–87%; Figure 4D) in accordance with the current European LeukemiaNet (ELN) risk stratification [26]. In contrast, *FLT3*-ITD together with wild type *NPM1* was associated with poor outcome (5-year EFS: 20%; CI: 11–32%; Figure 4D). Mutated *WT1* in NK-AML was associated with inferior 5-year EFS (30%; CI: 17–43%; Figure S1F), and co-occurrence of mutated *WT1* with *FLT3*-ITD further impacted prognosis (5-year EFS: 22%, CI: 9–39%; Figure 4E). Both mutated *CEBPA* (5-year EFS: 71%; CI: 53–83%) and mutated *GATA2* (5-year EFS: 85%; CI: 60–95%) were associated with favorable prognosis (Figure S1H,J). Importantly, considering the frequent co-occurrence of these mutations (Figure 3C), mutated *GATA2* remained associated with superior 5-year EFS independent of *CEBPA* mutational status (mutated *CEBPA*: 79%; CI: 47–93%; wildtype *CEBPA*: 100%) (Figure 4F). Of the remaining commonly mutated genes in NK-AML, neither mutations in *NRAS* (5-year EFS: 61%; CI: 43–74%), *PTPN11* (5-year EFS: 57%; CI: 43–59%), nor *TET2* (5-year EFS: 43%; CI: 43–74%), were associated with any significant differences in prognosis (Figures S1 and S2).

In a multivariate analysis stratified by mutational status, *FLT3*-ITDs were predictive of inferior EFS (HR: 1.77; CI: 1.17–2.69, *p* = 0.007) and OS (HR: 1.68; CI: 1.01–2.80, *p* = 0.47) adjusted for *NPM1* status (Table 2). Mutated *WT1* was also an independent predictor of poor outcome (EFS, HR: 1.87; CI: 1.21–2.88, *p* = 0.005) and OS (HR: 2.01; CI: 1.21–3.33, *p* = 0.007), and this effect was further strengthened by co-occurrence of *FLT3*-ITD (EFS, HR: 2.23; CI: 1.37–3.62, *p* = 0.001; OS, HR: 2.42; CI: 1.37–4.26, *p* = 0.002) irrespective of *FLT3*-ITD allelic ratio (Table 2). Mutated *NPM1* was an independent predictor of better EFS (HR: 0.21; CI: 0.12–0.39, *p* < 0.001) and OS (HR: 0.28; CI: 0.14–0.57, *p* < 0.001), including in co-occurrence with *FLT3*-ITD^low^ (EFS, HR: 0.20; CI: 0.05–0.81, *p* = 0.025). In the unadjusted analysis, mutated *CEBPA* was associated with better EFS and OS; however, after adjustment including *GATA2* status the effect was obliterated (Table 2). In contrast, we could confirm mutated *GATA2* as an independent predictor of favorable EFS (HR: 0.25; CI: 0.08–0.85, *p* = 0.026) adjusted for *CEBPA* status. In line with the 5-year survival estimates, mutated *NRAS*, *PTPN11*, and *TET2* were not predictive of disease outcome in the multivariate analysis.

## 4. Discussion

In this study, we exploited the publicly available data from the TARGET-AML study [10] to investigate the mutational landscape of pediatric NK-AML and its impact on prognosis. In NK-AML, the absence of any cytogenetic alterations further imposes the relevance of detailed molecular characterization to understand the genetic background of the disease, monitoring disease progression and to support informed risk stratification.

In 164 of 168 NK-AML patients not found to harbor any chromosomal aberrations from routine testing, the detailed sequencing analyses by WGS and TCS detected at least one protein-coding somatic mutation, demonstrating the utility of molecular characterization using these methods. Only four NK-AML patients were without any mutations identified.

Based on the results from WGS and TCS analysis we identified a unique mutational landscape in pediatric NK-AML characterized by a higher prevalence of mutated *CEBPA*, *FLT3*, *GATA2*, *NPM1*, *PTPN11*, *TET2*, and *WT1* as well as a lower prevalence of mutated *KIT*, *KRAS*, and *NRAS* compared with AK-AML (Figure 2 and Figure 3A,B). The commonly mutated genes were distributed across various TCGA functional groups compared to AK-AML. We noted that *TET2* and *IDH1-*2 that are involved in DNA methylation, and generally are considered to be genes associated with adult AML, constituted around 7% of the mutational landscape in pediatric NK-AML. We did, however, not find mutations in these three genes to be associated with older age within pediatric NK-AML (median: 12 years, range: 0–19, *p* = 0.613). Of the commonly mutated genes in NK-AML, *CEBPA*/*GATA2*, *FLT3*/*WT1*, and *FLT3*/*NPM1* were identified as frequently co-occurring events meanwhile mutated *CEBPA* was mutually exclusive of mutated *NPM1* and *FLT3*. This is of importance to consider when investigating the clinical implications of mutations in these genes.

Notably, six gene fusions not reported in the cytogenetic data were detected by WGS in NK-AML. This highlights the relevance of genomic sequencing to be implemented as clinical cytogenomic analyses as recently suggested [27,28]. Certain cryptic cytogenetic events of prognostic importance are difficult to capture in routine cytogenetic examinations, including, for instance, inv(16)(p13q24) (*CBFA2T3*-*GLIS2*) [29] and rearrangements involving 11p15 (*NUP98* fusions) [10]. For these cryptic events, WGS or transcriptome (RNA) sequencing may have a higher diagnostic yield detecting gene fusions or their expressed fusion transcripts, respectively [27,28,30,31].

In our multivariate analysis, we identified younger age, WBC count ≥50, *FLT3*-ITD, and mutated *WT1* as independent predictors of a poor outcome, and mutated *NPM1* and *GATA2* as independent predictors of favorable outcome in NK-AML. To our knowledge, younger age has not previously been highlighted as a risk factor in NK-AML. Possible confounders should be considered for this association, especially cryptic *CBFA2T3*-*GLIS2* fusions associated with poor prognosis, infancy, and FAB-M7 morphology (4 of 10 with known FAB class within the 0–1 year age group were M7 in this study) [32,33]. Nonetheless, this finding encourages further studies of the impact of age in independent cohorts. The clinical implications of *FLT3*-ITD with an inferior EFS is well established in the literature [11,12] and adverse outcomes related to mutated *WT1* have been reported [10,34,35]. The favorable impact on EFS of mutated *NPM1* irrespective of *FLT3*-ITD status has also previously been reported [36]. Mutated *CEBPA* has previously been reported to be an independent predictor of favorable outcome [14]; however, in our multivariate analysis, this association was obliterated following adjustment for *GATA2* status. In contrast, we identified mutated *GATA2* as an independent predictor of improved EFS adjusted for *CEBPA* status.

Some limitations must be considered interpreting these results. The complete dependency on the reported results of the study by Bolouri et al. may represent a limitation since we did not have any influence on the variant prioritization and a reanalysis of their raw data potentially could lead to the identification of previously unreported variants. Of note, we identified an older age among WGS/TCS analyzed subjects compared to non-sequenced subjects in the TARGET-AML cohort, possibly explained by the lower amount of bone marrow aspirate acquired from infant patients hindering inclusion for sequence analysis. This selection bias may have impacted the distribution of the mutational landscape of NK-AML as the prevalence of certain gene mutations, for instance, *NPM1*, did show some age dependency. Additionally, it should be noted that not all samples were analyzed using the same application. Hence, only 39 of 168 NK-AML were analyzed by WGS which resulted in an ascertainment bias in terms of limited detection of structural variation (e.g., gene fusions). Finally, the data used for this study did not allow us to adjust for the effect of stem cell transplantation in the multivariate analysis as stated in Section 2.4.

The TARGET-AML study [10] has proven the value of large-scale sequencing analysis to unravel the molecular background of a malignancy. With its comprehensive data, we have further described the genetics of NK-AML in comparison with AK-AML and its impact on prognosis. Such knowledge is essential in the clinic used for risk stratification and molecular minimal residual disease monitoring. Furthermore, the mutational landscape of NK-AML is also important to explore in relation to the emergence of several novel gene targeted therapies. These include, for instance, midostaurin for untreated *FLT3*-mutated AML currently undergoing a phase II trial [37], and inhibitors of mutated *IDH1*, such as ivosedinib that is Food and Drug Administration (FDA) approved for relapsed/refractory AML in children ≥12 years (NCT03245424).

Future molecular studies of pediatric AML will continue to be important to support this development. In this analysis, we only considered the genomics of NK-AML. However, to be able to obtain a more comprehensive understanding of the disease biology, multi-omics approaches investigating both the epigenome, transcriptome, microRNome, and proteome are needed.

## 5. Conclusions

Normal karyotype AML in children represents a unique group within pediatric AML with its own distinct genetic background and biology compared with abnormal karyotype AML. We identified younger age, WBC count ≥50, *FLT3*-ITD, and mutated *WT1* as independent predictors of a poor outcome and mutated *NPM1* and *GATA2* as independent predictors of favorable outcome.

## Figures and Tables

**Table 1:** Patient characteristics of normal and abnormal karyotype pediatric AML in the TARGET-AML study cohort.

	Abnormal Karyotype AML								Normal Karyotype AML	
	CBF-AML		*KMT2A* Rearranged AML		Other Abnormal Karyotype AML		All Abnormal Karyotype AML			
	n *	%	*n*	%	*n*	%	*n*	%	*n*	%
Patients	248	28	175	20	242	28	665	76	208	24
Sex (female)	115	46	92	53	115	48	322	48	92	44
Age (years), median (range)	11	(0–20)	3	(0–18)	8	(0–20)	9	(0–20)	12	(0–20)
0–1	15	6	69	39	61	25	145	22	14	7
2–9	86	35	55	31	76	31	217	33	61	29
10–19	147	59	51	29	105	43	303	46	133	64
WBC count (×10^9^/L), median (range)	35	(1−432)	43	(1−610)	24	(1−447)	32	(1−610)	31	(0−496)
CNS involvement	24	10	10	6	17	7	51	8	8	4
FAB classification										
M0	0		6	4	12	7	18	3	5	3
M1	16	7	5	3	30	17	51	9	45	27
M2	106	48	5	3	35	20	146	26	46	28
M4	101	45	26	17	31	18	158	29	36	22
M5	0		108	71	32	18	140	25	23	14
M6	0		0		7	4	7	1	6	4
M7	0		3	2	30	17	33	6	5	3
Not otherwise specified	4		3		23		30		16	
Unknown	21		19		42		82		26	
Genetics ^a^										
*NPM1* mutated	2/243	<1	1/170	<1	9/239	4	12/652	2	56/206	27
*CEPBA* mutated	1/245	<1	0/173		13/238	5	14/656	2	36/206	17
*FLT3*-ITD	10/247	4	4/173	2	43/242	18	57/662	9	85/208	41
*FLT3*-ITD low AR (<0.5)	6/247	2	1/173	<1	17/242	7	24/662	4	39/208	19
*FLT3*-ITD high AR (≥0.5)	4/247	2	3/173	2	26/242	11	33/662	5	46/208	22
SCT in CR1	3/243	1	24/164	15	55/213	26	82/620	13	46/176	26
Events										
Censored (no event)	154	62	68	39	95	39	317	48	101	49
IF, ID or death in CR	18	7	23	13	49	20	90	14	44	21
Relapse	76	31	84	48	98	41	258	39	63	30
Outcome % (95% CI)										
5-year EFS	62	(56–68)	38	(31–45)	38	(32–44)	47	(43–51)	48	(41–54)
5-year OS	80	(74–85)	58	(50–65)	50	(44–57)	64	(60–67)	62	(55–69)
5-year CIR	31	(25–36)	48	(41–55)	40	(34–47)	39	(35–43)	30	(24–37)
Included in the WGS/TCS analysis	202	81	127	73	164	68	493	74	168	81

* Listed numbers represent numbers and percentages unless otherwise stated. ^a^ Percentages based on samples tested. Abbreviations: AML, acute myeloid leukemia; AR, allelic ratio; CBF, core-binding factor; CIR, cumulated incidence of relapse; CNS, central nervous system; CR1, first complete remission; EFS, event-free survival; ID, induction death; IF, induction failure; ITD, internal tandem duplication; OS, overall survival; TCS, targeted capture sequencing; WBC, white blood cell; WGS, whole genome sequencing.

**Table 2:** Uni- and multivariate analysis of event-free and overall survival in NK-AML.

	Event-Free Survival							Overall Survival					
	Crude			Adjusted ^a^				Crude			Adjusted ^a^		
	5 y	HR	95% CI	HR	95% CI	*p* Value		5 y	HR	95% CI	HR	95% CI	*p* Value
**Overall analysis**													
Abnormal karyotype	47%	Ref.						64%	Ref.				
Normal karyotype	48%	1.01	(0.81–1.25)	0.95	(0.74–1.20)	0.648		62%	1.04	(0.80–1.34)	0.96	(0.72–1.27)	0.764
**NK-AML analysis**													
Female	53%	Ref.						63%	Ref.				
Male	44%	1.32	(0.90–1.95)	1.28	(0.86–1.90)	0.225		62%	1.12	(0.71–1.76)	1.10	(0.69–1.76)	0.692
Age 0–1 years	14%	2.21	(1.15–4.25)	2.72	(1.36–5.43)	0.005		21%	3.07	(1.51–6.22)	3.97	(1.87–8.46)	<0.001
Age 2–9 years	38%	Ref.						55%	Ref.				
Age 10–19 years	56%	0.59	(0.39–0.90)	0.60	(0.39–0.91)	0.018		70%	0.60	(0.37–0.99)	0.61	(0.37–1.01)	0.056
WBC count <50 × 10^9^/L	51%	Ref.						67%	Ref.				
WBC count ≥50 × 10^9^/L	42%	1.52	(1.04–2.23)	1.43	(0.96–2.12)	0.077		56%	1.68	(1.07–2.64)	1.70	(1.06–2.71)	0.028
Mutational status ^b^													
*FLT3*^wt^	50%	Ref.						62%	Ref.				
*FLT3*^mut (any)^ *	46%	1.09	(0.74–1.59)	1.21	(0.79–1.85)	0.377		62%	1.00	(0.64–1.57)	1.19	(0.71–1.99)	0.505
*FLT3*-ITD^neg^	53%	Ref.						73%	Ref.				
*FLT3*-ITD^pos^ ** †	40%	1.43	(0.98–2.09)	1.77	(1.17–2.69)	0.007		56%	1.31	(0.83–2.06)	1.68	(1.01–2.80)	0.047
with *FLT3*-ITD^low^	51%	1.06	(0.63–1.78)	1.49	(0.85–2.63)	0.165		60%	1.18	(0.65–2.13)	1.72	(0.89–3.32)	0.107
with *FLT3*-ITD^high^	31%	1.83	(1.18–2.83)	2.07	(1.29–3.33)	0.003		53%	1.43	(0.84–2.44)	1.64	(0.90–2.98)	0.107
*NPM1*^wt^	34%	Ref.						53%	Ref.				
*NPM1*^mut^ *	78%	0.21	(0.12–0.37)	0.21	(0.12–0.39)	<0.001		84%	0.25	(0.12–0.50)	0.28	(0.14–0.57)	<0.001
*NPM1*^wt^+*FLT3*-ITD	20%	2.82	(1.91–4.15)	3.06	(2.03–4.62)	<0.001		40%	2.34	(1.48–3.70)	2.64	(1.60–4.36)	<0.001
with *FLT3*-ITD^low^	29%	2.17	(1.26–3.74)	2.66	(1.50–4.72)	0.001		44%	2.10	(1.13–3.91)	2.69	(1.39–5.22)	0.003
with *FLT3*-ITD^high^	14%	3.00	(1.90–4.74)	3.08	(1.88–5.03)	<0.001		39%	2.20	(1.28–3.85)	2.34	(1.27–4.31)	0.006
*NPM1^mut^*+*FLT3*-ITD	78%	0.28	(0.12–0.65)	0.28	(0.12–0.64)	0.003		85%	0.29	(0.11–0.80)	0.30	(0.11–0.84)	0.022
with *FLT3*-ITD^low^	85%	0.19	(0.05–0.79)	0.20	(0.05–0.81)	0.025		85%	0.30	(0.07–1.22)	0.30	(0.07–1.28)	0.104
with *FLT3*-ITD^high^	69%	0.47	(0.17–1.29)	0.50	(0.18–1.37)	0.175		85%	0.35	(0.09–1.44)	0.38	(0.09–1.57)	0.179
*WT1*^wt^	59%	Ref.						73%	Ref.				
*WT1*^mut^ *	30%	1.85	(1.23–2.80)	1.87	(1.21–2.88)	0.005		47%	1.93	(1.19–3.13)	2.01	(1.21–3.33)	0.007
*WT1*^mut^+*FLT3*-ITD	22%	2.07	(1.30–3.32)	2.23	(1.37–3.62)	0.001		37%	2.18	(1.27–3.74)	2.42	(1.37–4.26)	0.002
with *FLT3*-ITD^low^	29%	1.90	(0.98–3.80)	2.39	(1.20–4.75)	0.013		42%	1.93	(0.91–4.08)	2.44	(1.11–5.35)	0.027
with *FLT3*-ITD^high^	16%	2.33	(1.26–4.29)	2.18	(1.15–4.12)	0.017		32%	2.41	(1.18–4.90)	2.37	(1.12–5.03)	0.025
*NRAS*^wt^	49%	Ref.						65%	Ref.				
*NRAS*^mut^ ***	61%	0.71	(0.41–1.25)	0.79	(0.45–1.41)	0.431		71%	0.78	(0.40–1.52)	0.86	(0.44–1.70)	0.666
*CEBPA*^wt^	42%	Ref.						58%	Ref.				
*CEBPA*^mut^ * ††	71%	0.41	(0.22–0.77)	0.78	(0.38–1.59)	0.495		81%	0.46	(0.22–0.96)	0.84	(0.34–2.07)	0.704
*PTPN11*^wt^	51%	Ref.						66%	Ref.				
*PTPN11*^mut^ ***	57%	0.90	(0.45–1.81)	1.18	(0.58–2.44)	0.647		71%	0.89	(0.38–2.08)	1.18	(0.49–2.81)	0.714
*GATA2*^wt^	48%	Ref.						63%	Ref.				
*GATA2*^mut^ *** ††	85%	0.23	(0.07–0.72)	0.25	(0.08–0.85)	0.026		90%	0.37	(0.12–1.19)	0.40	(0.12–1.31)	0.129
*TET2*^wt^	53%	Ref.						67%	Ref.				
*TET2*^mut^ ***	43%	1.33	(0.64–2.77)	1.81	(0.85–3.83)	0.440		55%	1.40	(0.60–3.28)	1.69	(0.71–4.03)	0.234

^a^ Adjusted for sex, age group, white blood cell count, and FLT3-ITD as appropriate. ^b^ Analysis includes genes mutated in ≥10 samples. † Adjusted regression model of *FLT3*-ITD mutational status included co-occurrence of *NPM1* mutation as covariate. †† Adjusted regression model of *GATA2* mutational status included co-occurrence of *CEBPA* mutation as covariate and vice versa. * Mutation status based on both genetic data from routine diagnostics and WGS/TCS analyses (n = 208). ** *FLT3*-ITD status is based on genetic data from routine diagnostics (n = 208). *** Mutation status based on genetic data from WGS/TCS analyses (n = 168). Abbreviations: CI, confidence interval; CR1, first complete remission; HR, hazard ratio; mut, mutation; NK-AML, normal karyotype acute myeloid leukemia; ref, reference; WBC, white blood cell; wt, wildtype.

## Data Availability

The publicly available data from the TARGET-AML study were obtained from the following webpage: https://target-data.nci.nih.gov/Public/AML/ (accessed on 24 January 2021).

## Supplementary Materials

The following are available online at https://www.mdpi.com/article/10.3390/genes12060792/s1, Figure S1: Additional Kaplan-Meier curves of event-free survival in pediatric normal karyotype AML; Figure S2: Kaplan-Meier curves of overall survival in pediatric normal karyotype AML.
